# Phenotypic and Genotypic Characterization of *NPRL2*-Related Epilepsy: Two Case Reports and Literature Review

**DOI:** 10.3389/fneur.2021.780799

**Published:** 2021-11-29

**Authors:** Yulin Sun, Lin Wan, Huimin Yan, Zhichao Li, Guang Yang

**Affiliations:** ^1^Department of Pediatrics, Chinese PLA General Hospital, Beijing, China; ^2^Department of Pediatrics, The First Medical Center, Chinese PLA General Hospital, Beijing, China; ^3^The Second School of Clinical Medicine, Southern Medical University, Guangzhou, China

**Keywords:** focal epilepsy, genotype, phenotype, *NPRL2*, case report

## Abstract

The phenotype of nitrogen permease regulator-like 2 (*NPRL2*) gene-related epilepsy clinically manifests as a range of epilepsy syndromes, including familial focal epilepsy with variable foci (FFEVF), sleep-related hypermotor epilepsy (SHE), temporal lobe epilepsy (TLE), frontal lobe epilepsy (FLE), and infantile spasms (IS). The association between phenotype and genotype of *NPRL2* variants has not been widely explored. This study aimed to explore the phenotype and genotype spectrum of *NPRL2*-related epilepsy. Here, we presented two clinical cases with *NPRL2*-related epilepsy, and discussed the characteristics, diagnosis, and treatment processes in the context of existing literature. Two novel *NPRL2* likely pathogenic variants were identified by next-generation sequencing, including one splicing mutation (c.933-1G>A), and one frameshift mutation (c.257delG). The results of literature review showed that there were a total of 20 patients with *NPRL2*-related epilepsy whose mutations were mostly missense and hereditary. These findings indicate that the possibility of *NPRL2* gene mutations in focal epilepsy should be considered for patients with family history, and that patients carrying different *NPRL2* variants have different clinical manifestations. Our study expanded the genotype spectrum of *NPRL2* and suggested that the type of *NPRL2* variants might provide important information for the prognosis evaluation.

## Introduction

Nitrogen permease regulator-like 2 (*NPRL2*), part of the GATOR1 complex that negatively regulates the mechanistic target of rapamycin (mTOR), reportedly affects brain development and function (1). The *NPRL2* gene, which is located on chromosome 3p21.3 and encodes a protein containing 380 amino acids, plays a key role of connecting the DEP domain-containing protein 5 (DEPDC5) and the nitrogen permease regulator-like 3 (NPRL3) of the GATOR1 complex (2). Pathogenic variants in the *NPRL2* gene can cause loss of function of the GATOR1 complex, thereby abnormally enhancing activity of the mTOR signaling pathway (3, 4). The phenotypic spectrum of *NPRL2*-related epilepsy ranges from sporadic early onset epilepsy, which is accompanied by cognitive impairment, to familial focal epilepsy, especially in the familial focal epilepsy with variable foci (FFEVF). These manifestations are similar to clinical phenotypes observed in patients with pathogenic variants in the *DEPDC5* and *NRPL3* genes (5). Here, we described the clinical characteristics of two cases with different novel likely pathogenic variants in *NPRL2*. Furthermore, we reviewed the advanced publications and summarized the associations between phenotype and genotype in *NPRL2*-related epilepsy.

## Methods

### Research Subjects

All procedures of this study were approved by the Ethics Committee of the Chinese PLA General Hospital (reference numbers: 2021-98). Written informed consent to participate in this study was provided by the legal guardian of the participants. Extracted patient data and medical records was deidentified and coded without identifiers. Our study involved one Chinese family clinically diagnosed with familial focal epilepsy (FFE) and one child clinically diagnosed with infantile spasms (IS). All participants were admitted to Chinese PLA General Hospital. Our inclusion criteria included (1) confirmed diagnosis of focal epilepsy and infantile spasms based on the criteria of the International League Against Epilepsy (ILAE) (6); (2) family history of epilepsy; and (3) pathogenic or likely pathogenic *NPRL2* mutation in the affected family members. All the probands received comprehensive clinical evaluations by experienced neurologists in our hospital. MRI (1.5T) and EEG were performed on these participants. Auxiliary examinations, including liver and kidney function, blood and urine organic acids, and electrolytes, were performed.

### Genetic Study

Two milliliters of venous blood of patient and parents were collected and DNA was extracted from the samples. Using Trio Whole-exome sequencing (WES) technology, it sequenced exons of nearly 20,000 human genes and about 20 bp upstream and downstream through targeted probe capture and high-throughput second generation sequencing (tested by Shenzhen Anji Kang Medical Inspection Laboratory). Bioinformatics analysis and mutation screening were based on original data from the sequencer, converted from.bcl to.fastq using bcl2fastq, and files were aligned with Human Reference Genome GRCH37/hg19 using BWA, SAMtools, and Picard software. The frequency of variation in the population was annotated by the international 1000 Genome Project, Genome Aggregation Database and Exome Aggregation Consortium. Online Mendelian Inheritance in Man (OMIM), Human Gene Mutation Database (HGMD), and disease-related human genome variation database (ClinVar) were used to annotate diseases. We used PolyPhen 2, SIFT, MutationTaster, and other software to predict and analyze protein function damage.

Pathogenic or likely pathogenic variants were screened according to the American College of Medical Genetics and Genomics (ACMG) classification guidelines and the clinical phenotypes of the patients (7). All clinically significant variations were validated by first-generation Sanger sequencing.

### Literature Search

A systematic literature search in PubMed and OMIM was performed. MeSH and title or abstract were used for all eligible studies that mainly focus on the *NPRL2* variants in FFE. The research strategy was as follows: “NPRL2” AND (“familial focal epilepsy” or “hereditary focal epilepsy” or “familial focal epilepsy with variable foci” or “familial epilepsy” or “infantile spasms”). Data from all eligible studies were analyzed and discussed by two reviewers.

## Results

### Case Presentation

#### Family A

A girl, aged 3 years and 11 months, presented with intermittent seizures mainly manifested by intermittent focal motor in the left limb or presented with generalized tonic-clonic seizure, with or without loss of consciousness. The seizures, which occurred during night sleep, mostly between 5 and 7 am, lasted for 15–20 s at a frequency of two to three times a week. The child was born of natural birth and had no history of asphyxia or hypoxia. Her intelligence, language, and movement were generally normal. Notably, her father had a history of focal epilepsy, which was diagnosed when he was 6 years old. His focal motor seizures have been under control with carbamazepine, although sensory auras may be triggered by drowsiness or fatigue. The grandfather of our index case also had a history of epilepsy, although the exact type of seizure was unknown. We found no evidence of abnormalities in the girl after comprehensive physical examination, which also included blood and urine organic acids, blood sugar, electrolyte, along with liver, and kidney function analysis. Moreover, head MRI showed no obvious signal abnormalities. However, EEG demonstrated medium and equal amount, short segment, paroxysmal, medium high potential, 2.2–2.9 HZ spike-slow wave, and sharp-slow wave discharges in the right anterior temporal region during sleep. The final diagnosis was temporal lobe epilepsy (TLE). She was orally administered with oxcarbazepine (32 mg/kg/d). The seizures were controlled, and she had acquired seizure freedom for 24 months by the end of June 2021.

#### Family B

A 4-month-old male infant had multiple unprovoked seizures 10 days after birth. The seizures were predominantly focal to generalized tonic-clonic seizures in semiology. These occurred more than 10 times a day and lasted for 10 s each. After oral phenobarbital treatment, the seizures were under control. During the drug reduction period, he developed epileptic spasms, presenting with sudden nodding with limbs extension. These occurred more than 10 times a day in clusters and, most often, shortly after waking. The child was born of natural birth and had no history of asphyxia or hypoxia. Family history was also normal. He could look up and turn over. There was no obvious developmental delay. After admission, we found no evidence of abnormalities in the auxiliary examinations. His head MRI showed no obvious signal abnormalities except that bilateral ventricles were enlarged. EEG demonstrated the typical findings of hypsarrhythmia. Disorganized, asymmetric, and asynchronous 150–200 μV slow waves could be seen in both wakefulness and sleep stages. Despite appropriate treatment with adrenocorticotropic hormone and topiramate (8 mg/kg/d), seizures remained uncontrolled by the end of September 2021.

### Novel Mutation in *NPRL2* Gene

**Figure 1:**
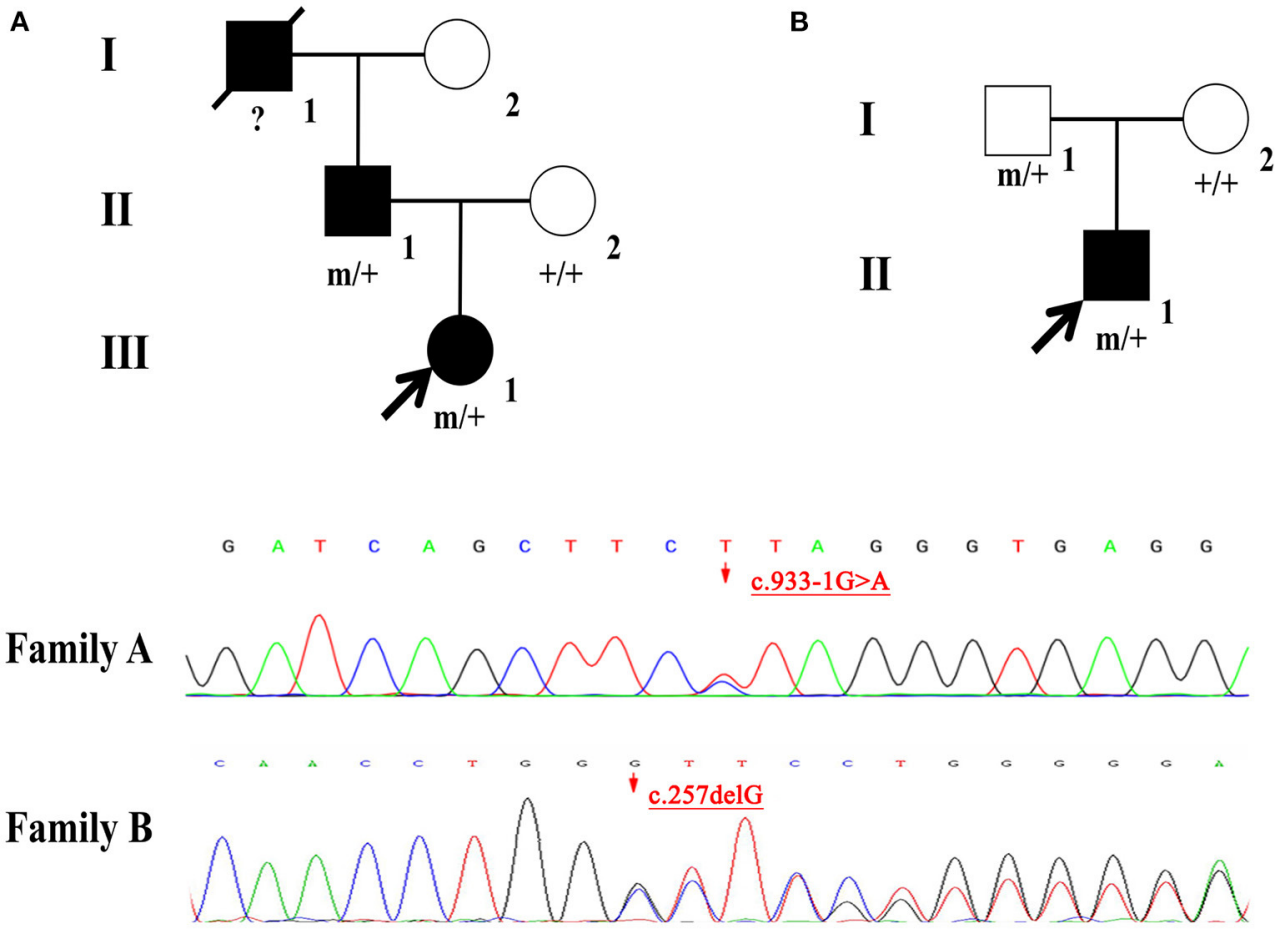
**(A,B)** Individuals carrying the NPRL2 mutation are indicated by “m/+”. Individuals with negative mutation are indicated by “+/+”, and individuals without blood samples are indicated by “?”.

Two novel mutations in the *NPRL2* (NM_006545.4), including c.933-1G>A and c.257delG, were identified by WES. Results from Sanger sequencing validated these findings and confirmed that both the father and child carried the same variant with a wild-type mother in Family A and B (Figure 1). The variant c.933-1G>A is a splicing mutation found in family A, which affects the structure of mRNA and results in loss of function mutation (LoF), meeting a highly pathogenic evidence (PVS1). This variant has neither been previously recorded in the reference population gene frequency database nor reported in the ClinVar and HGMD databases, meeting moderate intensity pathogenic evidence (PM2). The splicing variant c.933-1G>A was classified as likely pathogenic according to the ACMG criteria and guidelines (7). The variant c.257delG is a frameshift mutation found in family B, which can cause protein truncation or activate nonsense mediated mRNA degradation, thus affecting the function of coding protein products. Similarly, the variant c.257delG rating was described as PVS1 + PM2 and was classified as likely pathogenic.

## Discussion

The *NPRL2* gene, which is located on chromosome 3p21.3, with a transcript that is highly expressed in the brain frontal, temporal, parietal, and occipital lobes, has a similar distribution pattern to that of *DEPDC5* and *NPRL3* transcriptions (3, 8). *NPRL2* forms the GATOR1 complex together with DEPDC5 and NPRL3. *NPRL2* plays a crucial role in connecting DEPDC5 and NPRL3 and has also been implicated in development of hereditary focal epilepsy (8). GATOR1 dysfunction caused by any gene mutation of the above components can promotes activity of the mTOR signaling pathway, thereby causing hereditary focal epilepsy (9, 10). Other studies have proved that the Arg78 residue of *NPRL2* is a catalytic residue on GATOR1, and plays a role in regulating cell proliferation, differentiation, and death, as well as development and function of the brain by Rag GTPases-mediated activation of mTORC1 (11).

**Figure 2:**
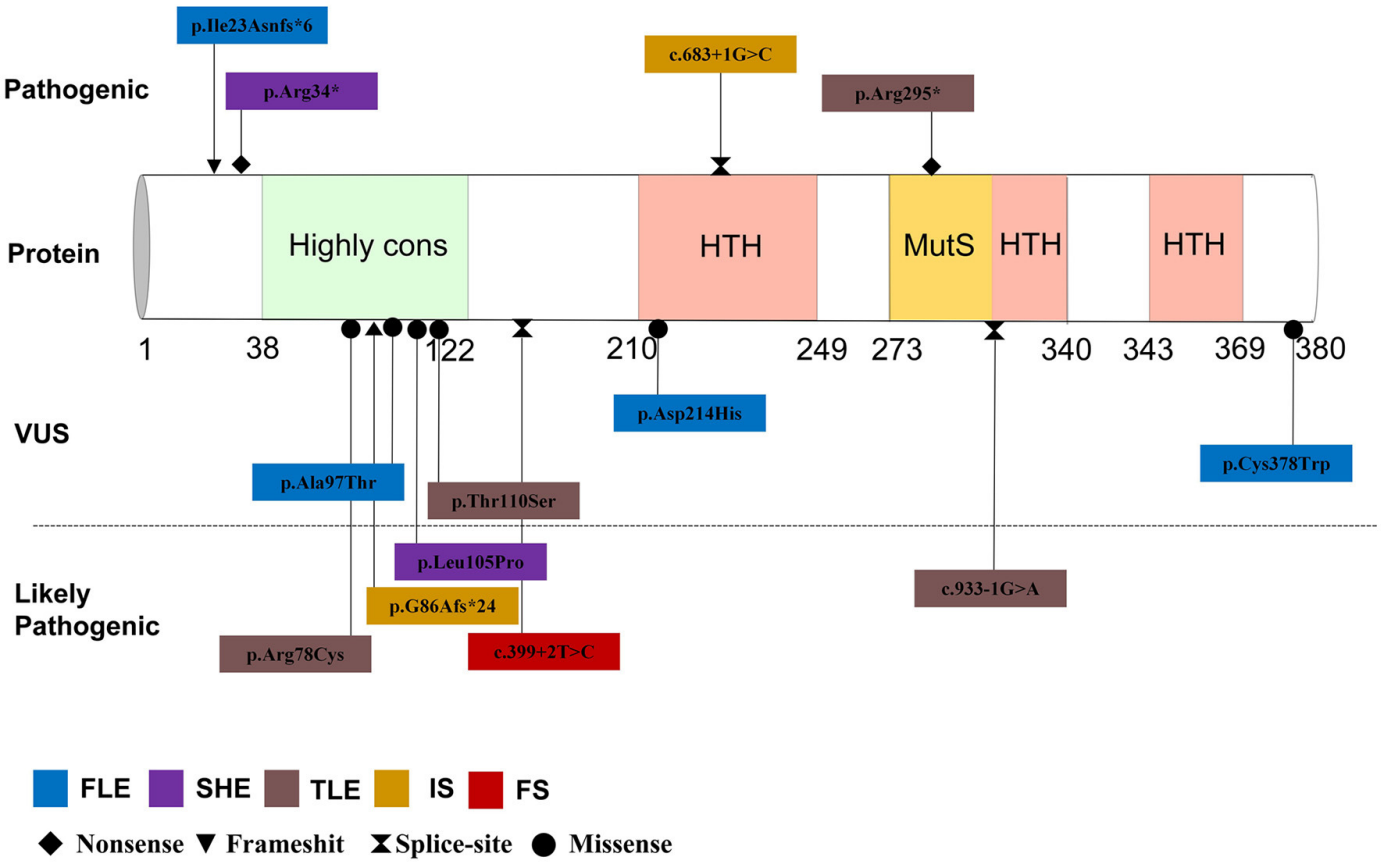
Mutation site and phenotype spectrum of NPRL2 gene.

By September 2021, a total of 22 patients with *NPRL2*-related epilepsy had been reported. Combined with our study, there were a total of 25 patients with *NPRL2*-related epilepsy, of which 11 were male and 13 were female, respectively, while the gender of one case was unknown (3, 8, 12–16). Among the cases whose specific clinical details could be obtained by manual screening, we found no evidence of abnormalities in their personal history, with all of them having normal development prior to seizure onset. Except for three cases of *de novo* variants and one case of unknown source of variant, the rest were familial genetic variation. Previous studies have shown that distribution of mutation types in the *GATORl* complex gene is mainly LoF mutations (nonsense and frameshift variants), and these account for 67% of all the cases (3). Combined with the reported cases, 13 *NPRL2* gene variants were detected, of which missense variants were the most common (46.2%), followed by splice (23.1%), nonsense (15.4%), and frameshift (15.4%) variants. Details of these variants and the corresponding phenotypes are shown in Figure 2 and Table 1.

**Table 1:** Genotypic and phenotypic characteristics of *NPRL2*-related epilepsy from previous literature identified to the present.

**References**	**Number of cases**	**cDNA variant**	**Protein alteration**	**Variant class**	**MRI**	**EEG discharge region**	**Phenotype**	**Number of AEDs used**	**Other treatments**	**Outcome**
Weckhuysen et al.	3	c.68_69delCT	p.Ile23Asnfs*6	Frameshift	N; PET: right frontoinsular hypometabolism/N/–	Right frontal island lobe and frontal orbital/left temporal region/–	FLE/TLE/UE/	8/1/-	Right-frontal orbitotomy/none/–	Controlled/controlled/–
Ricos et al.	2	c.100C>T	p.Arg34*	Nonsense	–	–	SHE	–	–	–
	1	c.883C>T	p.Arg295*	Nonsense	–	–	TLE	–	–	–
	1	c.329C>G	p.Thr110Ser	Missense	–	–	TLE	–	–	–
	1	c.640G>C	p.Asp214His	Missense	–	–	FLE	–	–	–
	5	c.314T>C	p.Leu105Pro	Missense	–	–	2SHE/1FE/2UE	–	–	–
Perucca et al.	2	c.232C>T	p.Arg78Cys	Missense	N/–	Left temporal region/–	TLE/UE	2/–	None/–	Uncontrolled/–
Baldassari et al.	1	c.100C>T	p.Arg34*	Nonsense	FCD in right frontal lobe	Right cingulate gyrus	SHE	8	Surgery, VNS	Controlled
	1	c.1134C>G	p.Cys378Trp	Missense	N; PET: N	Right frontal lobe	FLE	9	Ketogenic diet	Improved
	1	c.683+1G>C	p.(?)	Splice-site	FCD in left parieto-temporal	Hypsarrhythmia	IS	4	ACTH, surgery	Controlled
Deng et al.	1	c.289G>A	p.Ala97Thr	Missense	–	–	FLE	1	None	Controlled
Licchetta et al.	1	c.314T>C	p.Leu105Pro	Missense	–	–	SHE	–	–	–
Zhang et al.	2	c.399+2T>C	p.(?)	Splice-site	N/N	Left frontal and central regions/ left frontal region	FE; F to G T-C; IS/FS	2/–	–	Controlled/–
Our study	2	c.933-1G>A	p.(?)	Splice-site	N	Right anterior temporal region/–	TLE/FE	1/1	None	Controlled/controlled
	1	c.257delG	p.G86Afs*24	Frameshift	N	Hypsarrhythmia	IS	2	ACTH	Uncontrolled

*N, normal; –, not applicable or without information; FCD, focal cortical dysplasia; VNS, vagus nerve stimulation; ACTH, adrenocorticotropic hormone; SHE, sleep-related hypermotor epilepsy; TLE, temporal lobe epilepsy; FLE, frontal lobe epilepsy; IS, infantile spasm; FE, focal epilepsy; UE, unclassified epilepsy; FGTC, focal to generalized tonic-clonic; FS, febrile seizures*.

A total of 11 cases with age of onset can be obtained, which revealed that *NPRL2*-related epilepsy can occur from infancy to adulthood. Previous studies have found no significant relationship between the phenotype of epilepsy with affected components in the GATORl complex, which may be attributed to the co-performing function of the three protein composition complexes (2). Focal seizure was the most common type of seizure, although tonic seizures and epileptic spasms were also observed. The most common epileptic phenotype was SHE, which was observed in six cases, followed by TLE (five cases), FLE (four cases), and IS (two cases). Two cases of unclassified focal epilepsy (FE) and four cases of unclassified epilepsy (UE) were also included. One case presented multiple seizure types (FE, IS, and focal to generalized tonic-clonic seizures) and one case presented febrile seizures (FS) in the latest study (16).

Notably, a majority of *NPRL2* gene-associated epilepsy exhibited no structural damage, suggesting that the epileptogenic mechanisms may generate a relatively wide range of neurofunctional abnormalities and epileptogenic network formation (14). Eleven cases described the findings of MRI, of which eight patients with normal brain MRI. All three children with definite epileptogenic foci were surgically treated, with post-operative histological examination revealing focal cortical dysplasia (FCD) Ia, FCD IIa, and FCD Ia. On the other hand, brain MRI of one case showed no abnormalities, but head positron emission tomography (PET) demonstrated right frontoinsular hypometabolism. It suggested that PET may be helpful to detect focal structural abnormalities in patients with MRI-negative hereditary focal epilepsy.

Of the 25 patients included in this study, we obtained information about antiseizure medications in 11 cases, of which 6 (54.5%) were drug-resistant epilepsy. Among the five patients with drug-controlled seizures, two were treated with oxcarbazepine, one with sodium valproate, and one with a combination of topiramate and vitamin B6 therapy. Another case was controlled by carbamazepine, but there were obvious onset precursors in case of fatigue, insufficient sleep, and continuous missed medication. The underlying molecular mechanism suggests that mTOR inhibitors, such as rapamycin, may be promising drugs for *NPRL2* gene-related epilepsy, due to the abnormal activation of the mTOR signaling pathway caused by *NPRL2* gene variation, although further explorations are still needed to ascertain the stage of disease treatment (5, 17, 18).

## Conclusion

We reported two novel *NPRL2* likely pathogenic variants identified in two Chinese families. In patients with a family history of focal epilepsy, *NPRL2* variants should be considered. Combined with the reported cases, we found that patients harboring LoF mutations tend to suffer from refractory epilepsy and other severe comorbidities. Our study might provide significant information to physician for the treatment and prognosis evaluation of patients with *NPRL2*-related epilepsy.

## Data Availability Statement

The datasets presented in this article are not readily available due to ethical and privacy restrictions. Requests to access the datasets should be directed to the corresponding author.

## Ethics Statement

The studies involving human participants were reviewed and approved by the Ethics Committee of the Chinese PLA General Hospital. Written informed consent to participate in this study was provided by the participant's legal guardian/next of kin. Written informed consent was obtained from the individuals and/or minor's legal guardian/next of kin for the publication of any potentially identifiable images or data included in this article.

## Author Contributions

YS and LW wrote the first draft. HY and ZL revised the manuscript. GY drafted the manuscript and the final approval version to be published. All authors contributed to the article and approved the submitted version.

## Funding

This research was supported by grants from the Medical Big Data and Artificial Intelligence Research and Development Project of the Chinese PLA General Hospital [reference: 2019MBD-004] and Epilepsy Research Fund of China Association Against Epilepsy-UCB Fund [reference: CU-B-2021-11].

## Conflict of Interest

The authors declare that the research was conducted in the absence of any commercial or financial relationships that could be construed as a potential conflict of interest.

## Publisher's Note

All claims expressed in this article are solely those of the authors and do not necessarily represent those of their affiliated organizations, or those of the publisher, the editors and the reviewers. Any product that may be evaluated in this article, or claim that may be made by its manufacturer, is not guaranteed or endorsed by the publisher.
